# Supplementary figures and images for: RHEB/mTOR hyperactivity causes cortical malformations and epileptic seizures through increased axonal connectivity
Source: PLoS Biol. 2021 May 26;19(5):e3001279. doi: 10.1371/journal.pbio.3001279 (PMC8186814; doi:10.1371/journal.pbio.3001279)

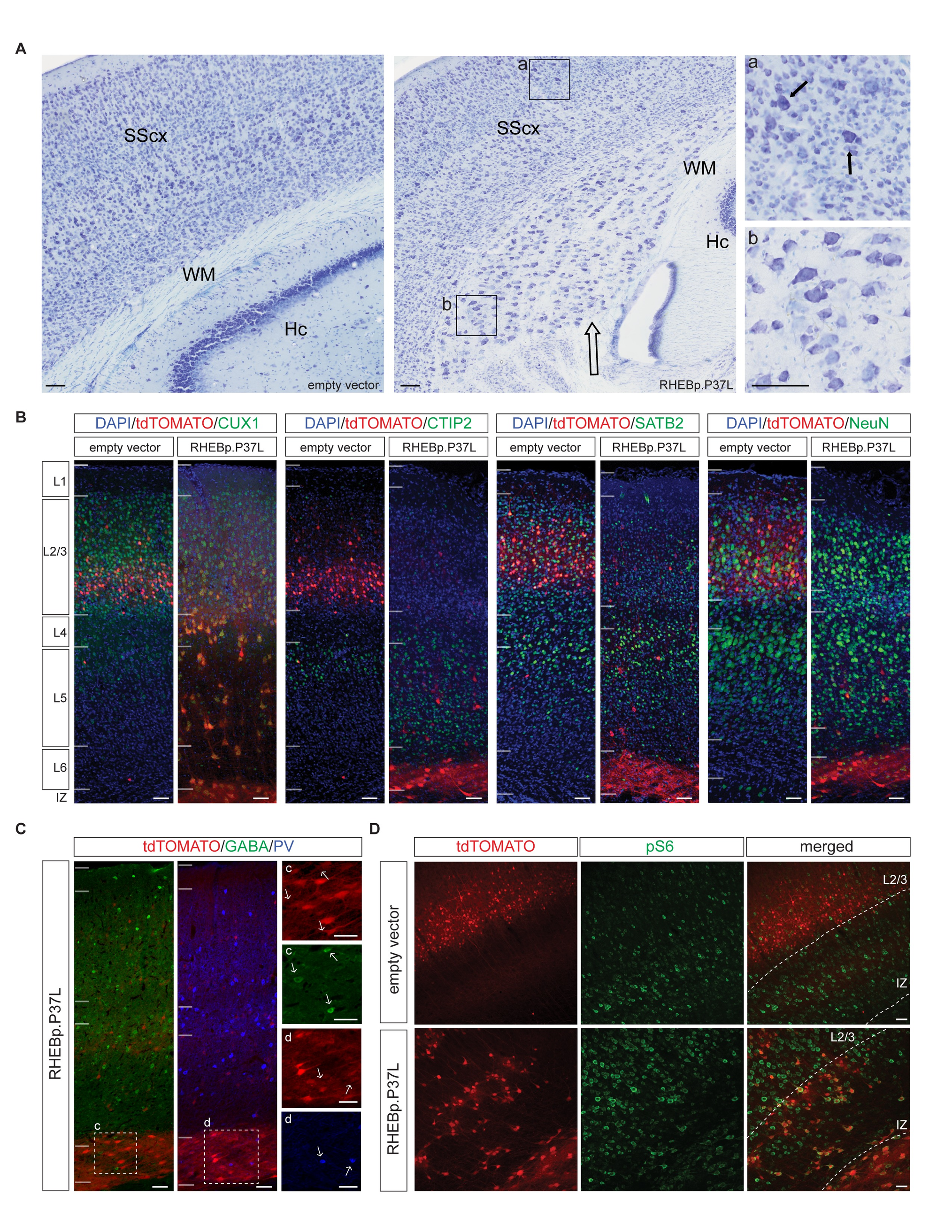

Supplement: S1 Fig — (A) Nissl staining of coronal brain sections from 5-week-old mice shows the presence of a clear heterotopia (indicated by the empty arrow) in the WM of RHEBp.P37L targeted SScx compared to the empty vector control situation. Boxes a and b represent magnifications of layer 2 (a) and the heterotopia (b), highlighting the targeted dysplastic and enlarged cells (indicated by the arrows); scale bars: 100 μm. (B) Representative overview images of coronal sections (SScx) of empty vector control and RHEBp.P37L targeted mice (5 weeks old) probed with common cortical layers markers CUX1 (L2/3 marker), CTIP2 (L5 marker), SATB2 (cortical projection neuron marker), or NeuN (mature neuron marker). (C) Representative images of coronal sections (SScx) of RHEBp.P37L targeted mice (5 weeks old) probed with GABA and PV markers for interneurons; magnification pictures of the heterotopia in (c) and (d) show that GABA- and PV-positive cells (indicated by the white arrows) are not positive for tdTomato (D) overview of the targeted SScx of empty vector control and RHEBp.P37L targeted mice stained for pS6-240, a readout of mTOR activity. Scale bars: 50 μm. Hc, hippocampus; IZ, intermediate zone; L2/3, layer 2/3; mTOR, mammalian target of rapamycin; SScx, somatosensory cortex; WM, white matter. (TIF) [file pbio.3001279.s001.tif]

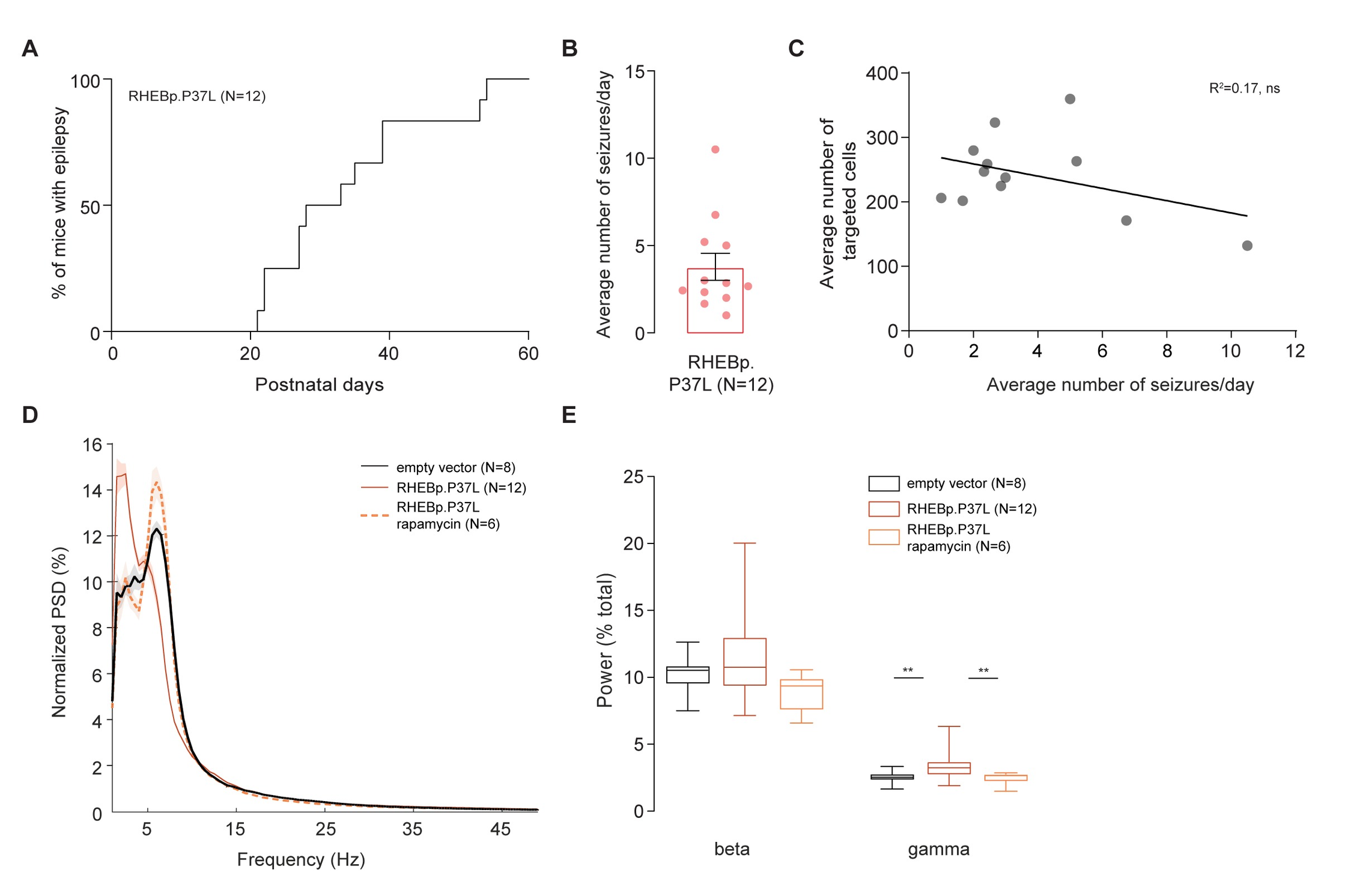

Supplement: S2 Fig — (A) Onset of seizure activity for the RHEBp.P37L group (mean ± SEM: 33.33 days ± 3.26; N indicates number of mice). (B) Average number of seizures per day of mice showing seizure activity measured with EEG until 9–12 weeks of age; each data point represents the average per mouse measured at least over 2 separate sessions of recordings (3 days each). (C) Simple scatter correlation graph with best fit regression line (Y = −9.5*X + 278.1), showing no correlation between the average number of targeted cells (measured over 3 anatomically matched nonconsecutive targeted slices per mouse) and the average number of seizures per animal shown in figure (B); r(10) = −0.41, p = 0.19, two-tailed Pearson’s correlation. (D) Extended normalized PSD shown in Fig 3E to include the beta and gamma frequencies (till 50 Hz); data are presented as mean (thick lines) ± SEM (shadows); N in the legend indicates number of mice per group. (E) Quantification of the beta (13–30 Hz) and gamma (30–50 Hz) frequency bands over the total power; box plots represent minimum and maximum value with median; N in the legend indicates number of mice per group. See S2 Table for statistics; the data underlying this figure can be found in S2 Data. **p < 0.01. EEG, electroencephalography; LFP, local field potential; ns, non-significant; PSD, power spectrum density. (TIF) [file pbio.3001279.s002.tif]

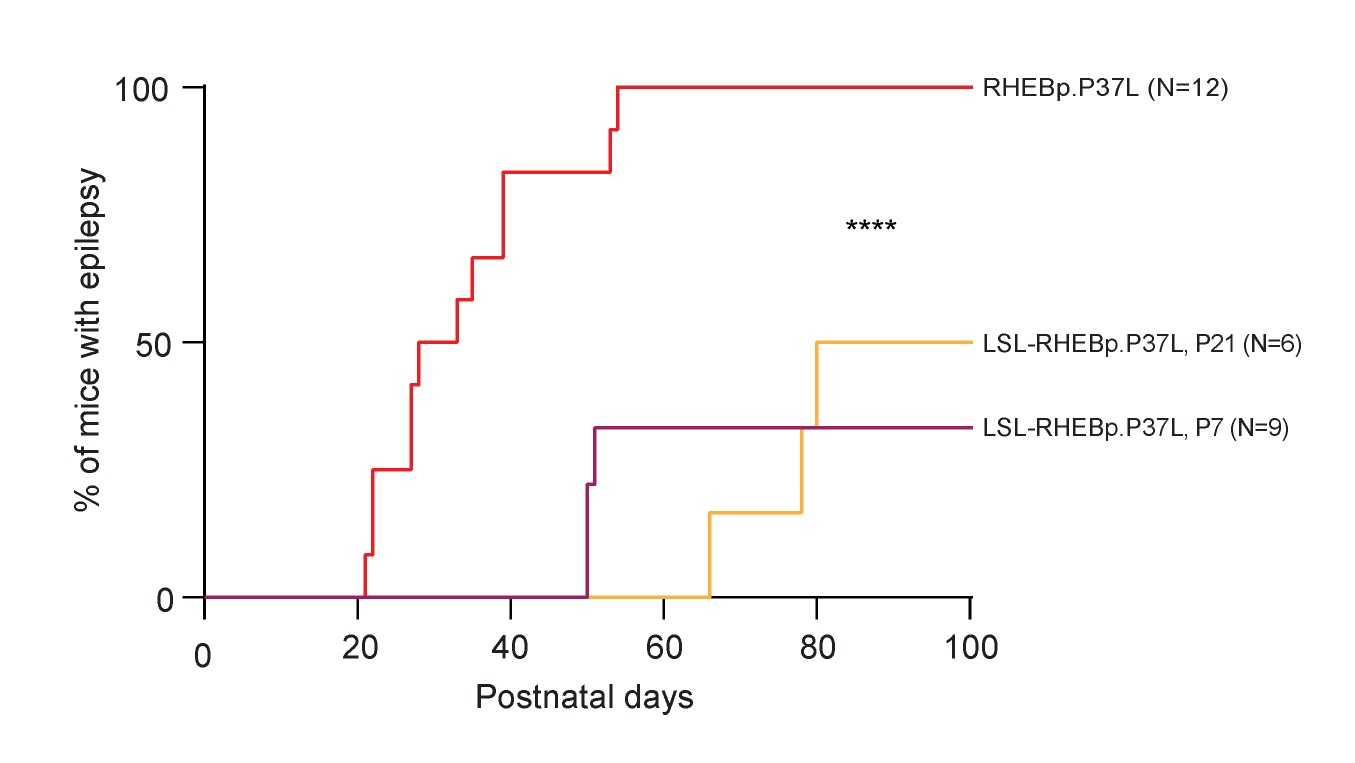

Supplement: S3 Fig — Onset of seizure activity for the LSL-RHEBp.P37L groups after treatment with tamoxifen (4 injections) starting at either P7 (purple line, mean ± SEM: 50 days ± 0) or at P21 (yellow line, mean ± SEM: 74.6 days ± 4.37) compared to the RHEBp.P37L group (red line) (chi-squared (2) = 25.33, p < 0.0001; log-rank test; N indicates number of mice). The data underlying this figure can be found in S4 Data. (TIF) [file pbio.3001279.s003.tif]

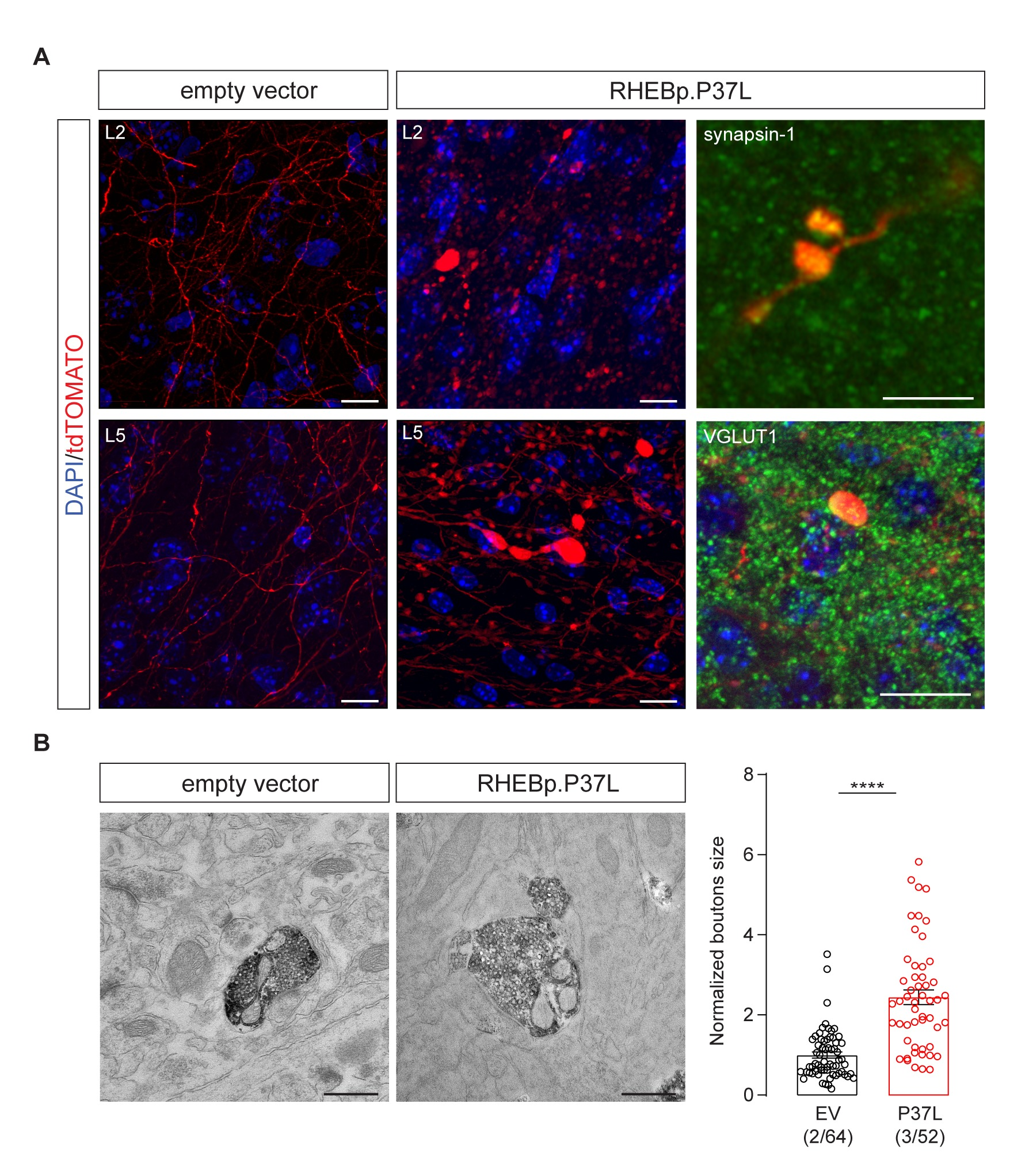

Supplement: S4 Fig — (A) Representative zoomed in pictures of the contralateral S1 (L2/3 and L5) of both control EV mice and RHEBp.P37L mice (P50); note the presence of enlarged terminals and boutons in RHEBp.P37L expressing cells that are positive for Synapsin-1 (a marker for synaptic vesicles, in green) and VGLUT1 (a marker for glutamatergic neurons, in green). Scale bars: 10 μm (overview), 5 μm (boutons). (B) Representative EM pictures of contralateral S1 boutons of control EV mice and RHEBp.P37L mice (P21) and quantification of the size, showing increase in size in the RHEBp.P37L mice (Mann–Whitney U = 465, p < 0.0001, ****, two-tailed Mann–Whitney test); numbers in the graph indicate number of animals/number of boutons analyzed. Scale bars: 500 nm. The data underlying this figure can be found in S5 Data. EV, empty vector; L2/3, layer 2/3. (TIF) [file pbio.3001279.s004.tif]

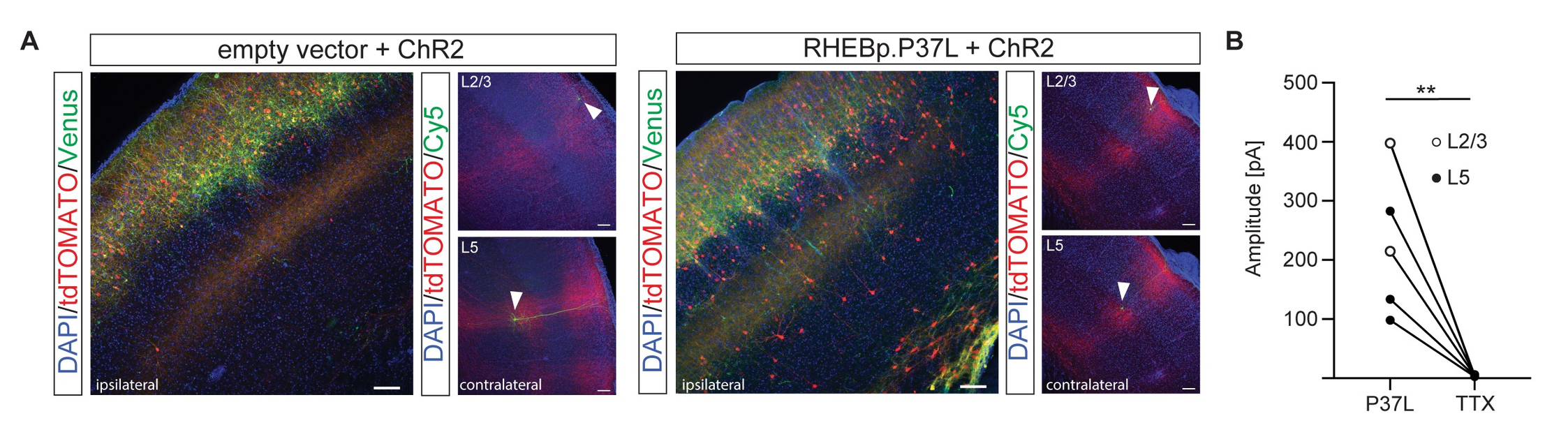

Supplement: S5 Fig — (A) Representative images showing expression of ChR2 (in green) and either EV (left) or RHEBp.P37L (right) constructs in red (tdTomato+ cell) on the ipsilateral targeted S1; examples of contralateral patched cells in either L2/3 or L5 filled with byocitin and stained with streptavidin-Cy5 are shown for each condition and indicated with arrowheads (note that for the contralateral pictures, ChR2-Venus is not shown and green represents byocitin-Cy5); scale bars: 100 μm. (B) Wash-in of TTX in RHEBp.P37L slices proves the action potential dependence of photostimulation evoked responses in L2/3 and L5; t(5) = 4.8, p = 0.005, two-tailed paired t test; **p < 0.01. The data underlying this figure can be found in S6 Data. ChR2, channelrhodopsin-2; EV, empty vector; TTX, tetrodotoxin. (TIF) [file pbio.3001279.s005.tif]

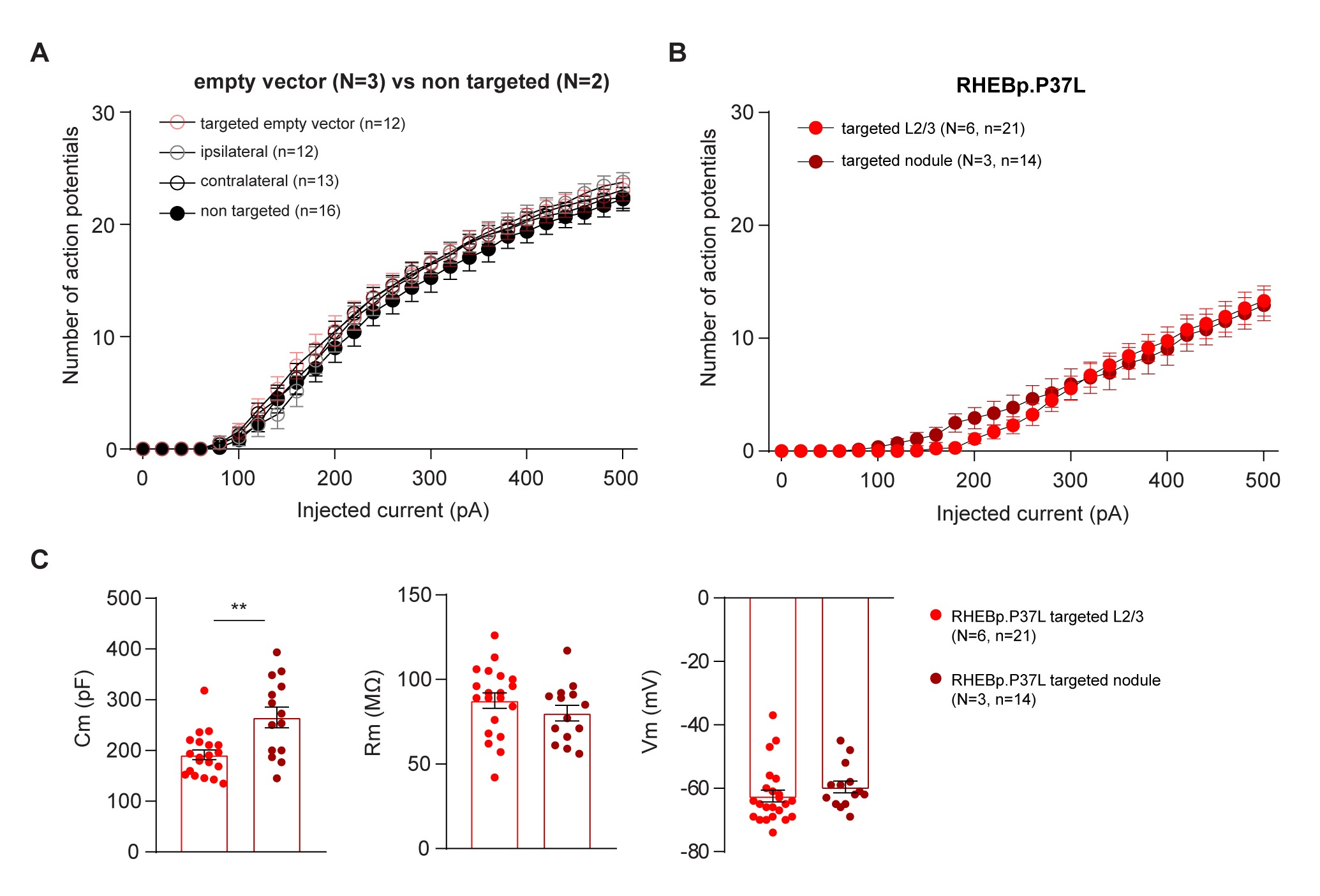

Supplement: S6 Fig — (A) Number of action potentials in response to increasing depolarizing currents shows that there is no difference in excitability in empty vector targeted mice or nontargeted mice; data are presented as mean ± SEM; interaction injected current/group condition: F (75, 1225) = 0.7275, nonsignificant; mixed-effects analysis; N = number of mice and n = number of cells analyzed. (B) Number of action potentials in response to increasing depolarizing currents shows that there is no difference in excitability in cells targeted with RHEBp.P37L based on their location (L2/3 and nodule); data are presented as mean ± SEM; interaction injected current/group condition: F (25, 824) = 0.95, nonsignificant; mixed-effects analysis; N = number of mice and n = number of cells analyzed. (C) Analysis of the passive membrane properties (Cm, Rm, and Vm) of pyramidal cells in L2/3 and cells in targeted cells in the nodule of RHEBp.P37L mice; note the increase in Cm of the targeted cells in the nodule, suggesting a bigger soma size compared to L2/3 cells; Cm: t(32) = 3.6; p = 0.001, two-tailed unpaired t test; Vm: t(35) = 1.02; p = 0.31, two-tailed unpaired t test; Rm: t(32) = 1.11; p = 0.28, two-tailed unpaired t test; numbers in the legend indicate number of targeted mice (N) and number of cells (n) analyzed; data are presented as mean ± SEM, and single data points indicate the values of each cell. The data underlying this figure can be found in S8 Data. Cm, capacitance; L2/3, layer 2/3; Rm, membrane resistance; Vm, resting membrane potential. (TIF) [file pbio.3001279.s006.tif]

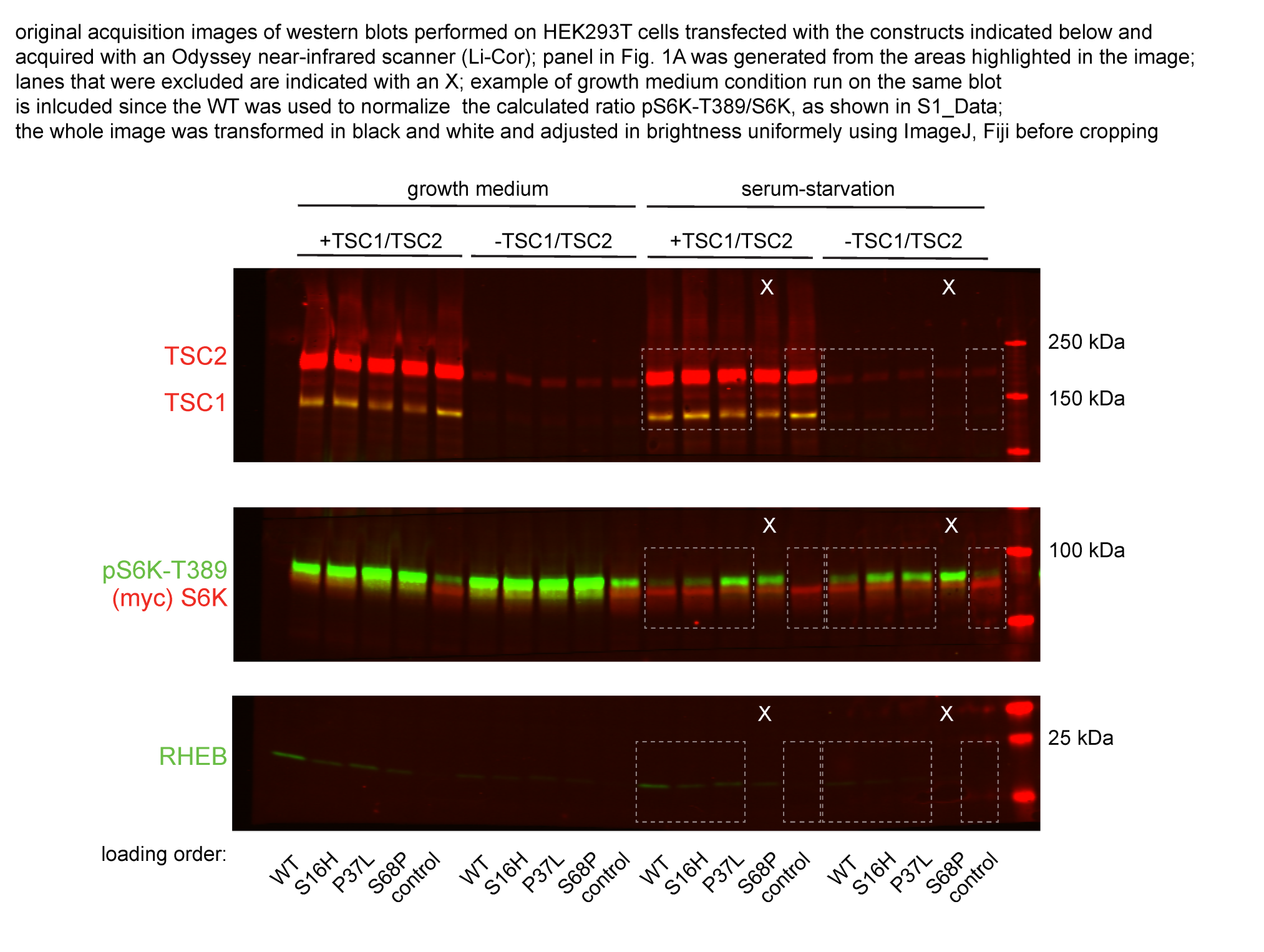

Supplement: S1 Raw Images — (TIF) [file pbio.3001279.s015.tif]
