# Supplementary material for: RHEB/mTOR hyperactivity causes cortical malformations and epileptic seizures through increased axonal connectivity
Source: PLoS Biol. 2021 May 26;19(5):e3001279. doi: 10.1371/journal.pbio.3001279 (PMC8186814; doi:10.1371/journal.pbio.3001279)
Supplement: S1 Table — The table summarizes the statistical tests and values obtained upon analysis of the data presented in Fig 1A. (DOCX) [file pbio.3001279.s016.docx]

| **S1 Table.** Statistical analysis related to Fig 1A | | | |
| --- | --- | --- | --- |
| **Test applied: Two-way ANOVA** | | | |
| **Source of variation** | **F (DFn, DFd)** | **P value** | **P value summary** |
| TSC -/+ | F (1, 35) = 13.38 | 0.0008 | *** |
| Group condition | F (3, 35) = 42.39 | <0.0001 | **** |
| Interaction | F (3, 35) = 2.377 | 0.0866 | ns |
| **Post hoc: Sidak’s multiple comparisons test** | | |  |
|  | **Comparison** | **Adjusted P value** | **P value summary** |
| TSC – *vs* TSC+ | RHEB wt | 0.0486 | * |
|  | RHEBp.S16H | 0.0049 | ** |
|  | RHEBp.P37L | 0.9791 | ns |
|  | Empty vector control | 0.9621 | ns |
| **Post hoc: Tukey’s multiple comparisons test** | | | |
|  | **Comparison** | **Adjusted P value** | **P value summary** |
| TSC – | RHEB wt vs RHEBp.S16H | 0.0205 | * |
|  | RHEB wt vs RHEBp.P37L | 0.0001 | *** |
|  | RHEB wt vs Empty vector control | 0.0151 | * |
|  | RHEBp.S16H vs RHEBp.P37L | 0.2859 | ns |
|  | RHEBp.S16H vs Empty vector control | <0.0001 | **** |
|  | RHEBp.P37L vs Empty vector control | <0.0001 | **** |
| TSC+ | RHEB wt vs RHEBp.S16H | 0.3696 | ns |
|  | RHEB wt vs RHEBp.P37L | <0.0001 | **** |
|  | RHEB wt vs Empty vector control | 0.7682 | ns |
|  | RHEBp.S16H vs RHEBp.P37L | 0.0009 | *** |
|  | RHEBp.S16H vs Empty vector control | 0.0603 | ns |
|  | RHEBp.P37L vs Empty vector control | <0.0001 | **** |

ns: non-significant, * *p*<0.05, ** *p*<0.01, *** *p*<0.001, **** *p*<0.0001
