# Supplementary material for: RHEB/mTOR hyperactivity causes cortical malformations and epileptic seizures through increased axonal connectivity
Source: PLoS Biol. 2021 May 26;19(5):e3001279. doi: 10.1371/journal.pbio.3001279 (PMC8186814; doi:10.1371/journal.pbio.3001279)
Supplement: S2 Table — The table summarizes the statistical tests and values obtained upon analysis of the data presented in Fig 2E and 2F and S2D and S2E Fig. (DOCX) [file pbio.3001279.s017.docx]

| **S2 Table.** Statistical analysis related to Fig 2E-F and S2D-E Fig | | | |
| --- | --- | --- | --- |
| **Test applied LFP: Two-way RM ANOVA** | | | |
| **Source of variation** | **F (DFn, DFd)** | **P value** | **P value summary** |
| Band Frequency | F (1.100, 53.92) = 353.7 | P<0.0001 | **** |
| Group condition | F (2, 49) = 1.011 | P=0.3714 | ns |
| Interaction  Band frequencies/  group condition | F (6, 147) = 7.456 | <0.0001 | **** |
| **Tukey’s multiple comparisons test** | **Comparison** | **Adjusted P value** | **P value summary** |
| delta | empty vector control vs. RHEBp.P37L | 0.0479 | * |
|  | empty vector control vs. RHEBp.P37L - rapamycin | 0.9614 | ns |
|  | RHEBp.P37L vs. RHEBp.P37L - rapamycin | 0.0856 | ns |
| theta | empty vector control vs. RHEBp.P37L | 0.0024 | ** |
|  | empty vector control vs. RHEBp.P37L - rapamycin | 0.8288 | ns |
|  | RHEBp.P37L vs. RHEBp.P37L - rapamycin | 0.0053 | ** |
| beta | empty vector control vs. RHEBp.P37L | 0.2043 | ns |
|  | empty vector control vs. RHEBp.P37L - rapamycin | 0.0637 | ns |
|  | RHEBp.P37L vs. RHEBp.P37L - rapamycin | 0.0071 | ** |
| gamma | empty vector control vs. RHEBp.P37L | 0.0039 | ** |
|  | empty vector control vs. RHEBp.P37L | 0.8465 | ns |
|  | empty vector control vs. RHEBp.P37L - rapamycin | 0.0016 | ** |
| **Test applied ratio theta/delta: Kruskal-Wallis test** | | | |
| **Kruskal-Wallis H** | **df** | **P value** | **P value summary** |
| 13.37 | 2 | 0.0012 | ** |
| **Dunn’s multiple comparisons test** | **Comparison** | **Adjusted P value** | **P value summary** |
| ratio theta/delta | empty vector control vs. RHEBp.P37L | 0.0169 | * |
|  | empty vector control vs. RHEBp.P37L - rapamycin | >0.9999 | ns |
|  | RHEBp.P37L vs. RHEBp.P37L - rapamycin | 0.0036 | ** |

ns: non-significant, * *p*<0.05, ** *p*<0.01, **** *p*<0.0001
