# Supplementary material for: RHEB/mTOR hyperactivity causes cortical malformations and epileptic seizures through increased axonal connectivity
Source: PLoS Biol. 2021 May 26;19(5):e3001279. doi: 10.1371/journal.pbio.3001279 (PMC8186814; doi:10.1371/journal.pbio.3001279)
Supplement: S3 Table — The table summarizes the statistical tests and values obtained upon analysis of the data presented in Fig 6. (DOCX) [file pbio.3001279.s018.docx]

| **S3 Table.** Statistical analysis related to Fig 6 | | | | |
| --- | --- | --- | --- | --- |
| **Test applied:**  **Two-tailed** **Mann-Whitney test** | | | | |
| **Parameter/**  **Layer** | **Unit** | **Mean ± SEM** | **Mann-Whitney U**  **P value** | **P value summary** |
| Amplitude  L2/3 | pA | control: 54.54±10.77  RHEBp.P37L: 356.80±54.06 | U = 7  p=0.0004 | *** |
| Amplitude  L5 | pA | control: 132.0±45.71  RHEBp.P37L: 365.70±97.59 | U = 34  p=0.0226 | * |
| Charge  L2/3 | pA*ms | control: 353.40±82.25  RHEBp.P37L: 3079.0±516.90 | U = 6  p=0.0003 | *** |
| Charge  L5 | pA*ms | control: 1552.0±816.10  RHEBp.P37L: 3658.0±1007.0 | U = 36  p=0.0308 | * |
| Vm  L2/3 | mV | control: -77.83±1.50  RHEBp.P37L: -76.43±2.22 | U = 51.50  p=0.9851 | ns |
| Vm  L5 | mV | control: -73.0±0.55  RHEBp.P37L: -73.53±1.16 | U = 70  p=0.7933 | ns |
| R series  L2/3 | MΩ | control: 11.97±0.94  RHEBp.P37L: 11.88±0.71 | U = 51  p=0.9576 | ns |
| R series  L5 | MΩ | control: 14.91±1.11  RHEBp.P37L: 12.46±0.86 | U = 47  p=0.1289 | ns |
| Rm  L2/3 | MΩ | control: 105.50±10.92  RHEBp.P37L: 97.08±8.69 | U = 45  p=0.6324 | ns |
| Rm  L5 | MΩ | control: 131.50±22.75  RHEBp.P37L: 130.70±8.22 | U = 57  p=0.3383 | ns |

ns: non-significant, * *p*<0.05, *** *p*<0.001
