# Supplementary material for: RHEB/mTOR hyperactivity causes cortical malformations and epileptic seizures through increased axonal connectivity
Source: PLoS Biol. 2021 May 26;19(5):e3001279. doi: 10.1371/journal.pbio.3001279 (PMC8186814; doi:10.1371/journal.pbio.3001279)
Supplement: S4 Table — The table summarizes the statistical tests and values obtained upon analysis of the data presented in Fig 8B and 8C. (DOCX) [file pbio.3001279.s019.docx]

| **S4 Table.** Statistical analysis related to Fig 8B-C | | | | | | | | | | | |
| --- | --- | --- | --- | --- | --- | --- | --- | --- | --- | --- | --- |
| **Test applied on basic properties: One-way ANOVA** | | | | | | | | | | | |
| **Basic properties** | **F (DFn, DFd)** | | **P value** | | | | | **P value summary** | | | |
| Cm | F (3, 111) = 6.525 | | P=0.0004 | | | | | *** | | | |
| Rm | F (3, 111) = 10.47 | | P<0.0001 | | | | | **** | | | |
| Vm | F (3, 111) = 0.3580 | | P=0.7834 | | | | | ns | | | |
| **Post hoc: Tukey’s multiple comparisons test** | | | | | | | | | | | |
| **Comparison** | **Cm Adjusted P Value** | **Cm Summary** | **Rm Adjusted P Value** | | | **Rm Summary** | | **Vm Adjusted P Value** | | | **Vm Summary** |
| control vs. contralateral RHEBp.P37L | 0.9218 | ns | 0.9970 | | | ns | | 0.9028 | | | ns |
| control vs. ipsilateral RHEBp.P37L | 0.1568 | ns | 0.6241 | | | ns | | 0.9974 | | | ns |
| control vs. targeted RHEBp.P37L | 0.0003 | *** | <0.0001 | | | **** | | 0.9585 | | | ns |
| contralateral RHEBp.P37L vs. ipsilateral RHEBp.P37L | 0.6314 | ns | 0.6506 | | | ns | | 0.9751 | | | ns |
| contralateral RHEBp.P37L vs. targeted RHEBp.P37L | 0.0171 | * | <0.0001 | | | **** | | 0.7394 | | | ns |
| ipsilateral RHEBp.P37L vs. targeted RHEBp.P37L | 0.2425 | ns | 0.0033 | | | ** | | 0.9337 | | | ns |
| **Test applied on excitability (RHEBp.P37L vs control): Mixed-effects model analysis** | | | | | | | | | | | |
| **Source of variation** | **F (DFn, DFd)** | | | | **P value** | | | | **P value summary** | | |
| Injected current | F (2,770, 265.6) = 1228 | | | | <0.0001 | | | | **** | | |
| Group condition | F (3, 96) = 44.64 | | | | <0.0001 | | | | **** | | |
| Interaction current/condition | F (75, 2397) = 29.02 | | | | <0.0001 | | | | **** | | |
| **Post hoc: Tukey’s multiple comparisons test** | | | | | | | | | | | |
| **Main effect: group condition** | | | | **Mean difference** | | | **Adjusted P Value** | | | **P value summary** | |
| targeted RHEBp.P37L vs. ipsilateral RHEBp.P37L | | | | 7.349 | | | <0.0001 | | | **** | |
| targeted RHEBp.P37L vs. contralateral RHEBp.P37L | | | | -0.2674 | | | <0.0001 | | | **** | |
| targeted RHEBp.P37L vs. control | | | | -2.948 | | | <0.0001 | | | **** | |
| ipsilateral RHEBp.P37L vs. contralateral RHEBp.P37L | | | | -7.616 | | | <0.0001 | | | **** | |
| ipsilateral RHEBp.P37L vs. control | | | | -10.3 | | | 0.9447 | | | ns | |
| contralateral RHEBp.P37L vs. control | | | | -2.681 | | | <0.0001 | | | **** | |

ns: non-significant, * *p*<0.05, ** *p*<0.01, *** *p*<0.001, **** *p*<0.0001
