# Supplementary material for: RHEB/mTOR hyperactivity causes cortical malformations and epileptic seizures through increased axonal connectivity
Source: PLoS Biol. 2021 May 26;19(5):e3001279. doi: 10.1371/journal.pbio.3001279 (PMC8186814; doi:10.1371/journal.pbio.3001279)
Supplement: S5 Table — The table summarizes the statistical tests and values obtained upon analysis of the data presented in Fig 8E and 8F. (DOCX) [file pbio.3001279.s020.docx]

| **S5 Table.** Statistical analysis related to Fig 8E-F | | | | | | | | | |
| --- | --- | --- | --- | --- | --- | --- | --- | --- | --- |
| **Test applied on basic properties: One-way ANOVA** | | | | | | | | | |
| **Basic properties** | **F (DFn, DFd)** | | | | **P value** | | **P value summary** | | |
| Cm | F (3, 69) = 10.43 | | | | P<0.0001 | | **** | | |
| Rm | F (3, 69) = 16.01 | | | | P<0.0001 | | **** | | |
| Vm | F (3, 73) = 0.8526 | | | | P=0.4697 | | ns | | |
| **Post hoc: Tukey’s multiple comparisons test** | | | | | | | | | |
| **Comparison** | **Cm Adjusted P Value** | | **Cm Summary** | **Rm Adjusted P Value** | | **Rm Summary** | **Vm Adjusted P Value** | **Vm Summary** | |
| targeted RHEBp.P37L vs.  targeted RHEBp.P37L/LSL-TeTxLC | 0.1347 | | ns | 0.7179 | | ns | 0.6715 | ns | |
| targeted RHEBp.P37L vs.  contralateral RHEBp.P37L/LSL-TeTxLC | 0.0087 | | ** | 0.0004 | | *** | 0.9996 | ns | |
| targeted RHEBp.P37L/LSL-TeTxLC vs. contralateral RHEBp.P37L/LSL-TeTxLC | <0.0001 | | **** | <0.0001 | | **** | 0.7032 | ns | |
| targeted RHEBp.P37L/LSL-TeTxLC vs. contralateral RHEBp.P37L | 0.0006 | | *** | <0.0001 | | **** | 0.6425 | ns | |
| contralateral RHEBp.P37L/LSL-TeTxLC vs. contralateral RHEBp.P37L | 0.5782 | | ns | 0.9675 | | ns | 0.9971 | ns | |
| targeted RHEBp.P37L vs.  targeted RHEBp.P37L/LSL-TeTxLC | 0.1347 | | ns | 0.7179 | | ns | 0.6734 | ns | |
| **Test applied on excitability: Mixed-effects model analysis** | | | | | | | | | |
| **Excitability RHEBp.P37L/**  **LSL-TeTxLC** | | **F (DFn, DFd)** | | | | | **P value** | | **P value summary** |
| Injected current | | F (2.357, 251.5) = 870.7 | | | | | <0.0001 | | **** |
| Group condition | | F (4, 107) = 3714 | | | | | <0.0001 | | **** |
| Interaction current/condition | | F (100, 2667) = 21.99 | | | | | <0.0001 | | **** |
| **Post hoc: Tukey’s multiple comparisons test** | | | | | | | | | |
| **Comparison** | | **Mean difference** | | | | | **Adjusted P Value** | | **P value summary** |
| control vs.  targeted RHEBp.P37L/LSL-TeTxLC | | 6.556 | | | | | <0.0001 | | **** |
| control vs.  contralateral RHEBp.P37L/LSL-TeTxLC | | -0.3674 | | | | | 0.9490 | | ns |
| targeted RHEBp.P37L vs.  targeted RHEBp.P37L/LSL-TeTxLC | | -0.7935 | | | | | 0.4371 | | ns |
| targeted RHEBp.P37L vs.  contralateral RHEBp.P37L/LSL-TeTxLC | | -7.717 | | | | | <0.0001 | | **** |
| contralateral RHEBp.P37L vs.  targeted RHEBp.P37L/LSL-TeTxLC | | 9.504 | | | | | <0.0001 | | **** |
| contralateral RHEBp.P37L vs.  contralateral RHEBp.P37L/LSL-TeTxLC | | 2.581 | | | | | 0.0002 | | *** |

ns: non-significant, ** *p*<0.01, *** *p*<0.001, **** *p*<0.0001
