# Supplementary material for: RHEB/mTOR hyperactivity causes cortical malformations and epileptic seizures through increased axonal connectivity
Source: PLoS Biol. 2021 May 26;19(5):e3001279. doi: 10.1371/journal.pbio.3001279 (PMC8186814; doi:10.1371/journal.pbio.3001279)
Supplement: Attachment — Submitted filename: Response to Reviewers.doc [file pbio.3001279.s021.doc]

***Reviewer #1****: The manuscript biologically presented epileptogenesis of RHEB-p.P37L-derived cortical cells. Hyperactive mTOR-system is well-known as a pathogenesis of FCD, HME and other brain malformation with intractable epilepsy. The authors showed the evidence of abnormal cell to cell connection and autonomous excitability. This model will be useful for understanding the pathogenesis and therapeutic hint of various brain malformation-related epilepsy.*

We thank the Reviewer for the positive remarks on the value of our RHEBp.P37L mouse model for a better understanding of MCD pathogenesis. *I have to require the following correction and consideration.*

*1. As you know, malformed brain usually demonstrates abnormal interneuron distribution. And, the balance of excitatory neuron and inhibitory interneuron is sometimes discussed. You showed cell autonomous epileptogenesis of RHEB-p.P37L-cortical cells. RHEB-p.P37L-cortical cells might be speculated to be excitatory neurons. Therefore, I would like to request to confirm whether RHEB-p.P37L-cortical cells were excitatory neurons or GABAergic interneurons. Then, I additionally request the distribution of GABAergic interneurons in the pathological region.*

We thank the Reviewer for raising this point regarding the distribution of interneurons in the pathological context and their role in MCD.

To respond first to the question, **are the RHEBp.P37L-cortical cells excitatory neurons**: the migration of excitatory cortical cells born in the ventricular zone follows a radial pattern along the radial glia. Interneurons, on the other hand, are primarily born from the ganglionic eminence and follow a tangential migration pattern to reach the cortex. We performed *in utero* electroporation at E14.5, placing the plus pole of the electrodes in such way on the cortex that progenitor cells giving rise to L2/3 pyramidal cells are targeted (see [1]). To confirm the nature of the targeted cells, we performed immunostainings with Cux1, a marker for layer 2/3 neurons that is primarily expressed in glutamatergic projection neurons and only in a subpopulation of interneurons ([2,3]), and Satb2, which is exclusively expressed in excitatory projection cells ([4]). We calculated that approximately 80% of the RHEBp.P37L positive cells are also positive for Satb2, therefore excitatory (see **Figure 1F** and **Figure S1B** for an overview). In addition, we now also performed a staining with GABA and PV, markers for interneurons, and found no overlap between the RHEBp.P37L cells and the interneuron staining, confirming that these cells are not interneurons (**Figure S1C**).

Regarding **the distribution of interneurons in the pathological region**: While, as noted by Reviewer #1, abnormalities in the distribution of interneurons have been observed in FCD and lissencephaly in humans, this disturbance might occur as a secondary effect due to an abnormal cortical development of excitatory neurons ([5]). We have now analyzed the distribution of interneurons in our model and found that overall, the distribution of interneurons in the targeted cortex, and specifically of PV+ cells that represent the majority of cortical interneurons ([6]), is not affected (see *Rebuttal Figure 1*, provided below).

**Rebuttal Figure 1.** *Upper panel*: representative pictures of the targeted somatosensory cortex of a control mouse (left) and RHEBp.P37L mouse (right) stained for PV (in blue); dotted lines indicate the border of the intermediate zone (IZ, bottom) and delineate L2/3. ­­­Scale bars: 50 μm
*Lower panel*: distribution of PV+ cells in the targeted somatosensory cortex of empty vector mice (N=2, 5 weeks old) and RHEBp.P37L mice (N=3, 5 weeks old) calculated as % of all PV+ cells across the cortical layers, with bin1 indicating the upper layer 1 and bin 10 the lower layer 6. Data are presented as mean ± SEM.

However, in agreement with [5], we do find non-targeted GABA-positive neurons to be present in the heterotopic nodule (*Rebuttal Figure 2*, below). This indicates that the interneurons are indeed recruited to the nodule, which potentially is a way for the brain to try to keep the balance between inhibition and excitation. We have now included the GABA and PV stainings in **Figure S1 (S1C)** of the supplementary figures. If needed, we can also add the above analysis of the distribution of the interneurons.

**Rebuttal Figure 2.** Representative picture of the heterotopic nodule of a RHEBp.P37L mouse stained for GABA, showing the presence of interneurons in the nodule (in green). ­­Note that GABA positive cells are not positive for tdTomato (in red), therefore these represent non-targeted neurons. Scale bars: 50 μm

*2. I cannot understand Figure 3D. The number of tdTOMATO+ cells in the no-seizure cortex showed less than that in the seizure cortex in the figure panels. However, in the graph the average %cells in the no-seizure cortex showed less than that in the seizure cortex. I feel the results were discrepancy.*

We apologize for the confusion that this figure created. **Figure 3D** presents the data shown in **Figure 3B** but now separating the results of the cortical migration according to the presence (seizures) or absence (no seizures) of seizures for each mouse, correlating the degree of migration deficit with the presence of seizures. We have now clarified this in the results section of the manuscript text. The quantification shown is the percentage of the total number of targeted cells that manage to reach layer 2/3 per mouse (y axis in the graph). As visible from the representative images, this percentage is on average higher in the mice that do not show seizures, compared to mice that do show seizures. Indeed, almost 100% of the targeted cells in the ‘no seizure’ condition is located in L2/3 while for the ‘seizure’ condition a certain percentage of targeted cells can be found in deeper layers of the cortex, unable to migrate out. We now modified the y axis label from ‘*average % cells L2/3*’ into *‘% targeted cells L2/3 per mouse*’ to keep the consistency with the y axis label in **Figure 3B** and included a more detailed description of the data presented in **Figure 3D** in the legend.

***Reviewer #2****: Authors present a study focused on mTOR activator RHEB-related MCD. They investigated an IUE mouse model (previously published) expressing the p.P37L variant and show it resembles the human pathology with migration defects, mTOR activation and epilepsy.
Authors address key questions in the field on the cause of epileptogenesis and the role of cortical malformations in seizures generation. Among novel finding, they show that RHEB P37L expressing mutant are hypoexcitable, but lead to increase excitability in contralateral neurons due to abnormal axonal connectivity and neurotransmitter release.

Some data are not novel (neuronal migration defect and spontaneous tonic-clonic seizures in the RHEBp.P37L IUE mouse model for instance). I suggest to better highlight the novel aspects of the study such as abnormal axonal connectivity leading to hyperexcitability of distal neurons.*

We thank the reviewer for this suggestion. We are indeed aware that some of our data confirm previous findings obtained with other models of MCD and MCD-related epilepsy, and we believe that it is important to stress the commonalities between these models and to highlight the fact that different mouse models show similar outcome despite the differences in methodology and/or genetic background. Following the reviewer’s suggestion, we now emphasized more clearly the novel aspects of our study.

*Suggestions for improvement:
- In several parts, a better clarification of which brain area is investigated should be provided. For instance, linking the targeted cells to EEG and LFP recordings (please precise ipsi/contralateral side).*

We thank the reviewer for noticing this. Where necessary, a clarification of the brain area or cortical layer under investigation is now specified throughout the text.

For EEG recordings we placed the electrodes bilaterally on the SScx with the intent to detect the epileptic events as shown in **Figure 2B** and **C** and count the number of seizures per day. Since these measures do not have very high resolution and the epileptic activity was seen in all channels, we did not make a distinction between the ipsi- or contralateral side in counting the number of seizures.

The LFP recordings were done by placing the electrodes also bilaterally in the SScx at the depth of layer 2/3. In this case, we did analyze the frequency dynamics of cortical brain activity separately for the targeted (ipsilateral) and non-targeted (contralateral) SScx, to observe if there was any difference in brain activity that we could link to the targeted area. However as we also stated in the text: “The normalized averaged power spectrum of the RHEBp.P37L group did not reveal a significant difference between the ipsilateral and contralateral (non-targeted) SScx (targeting: F(1, 22)=1.43, p=0.25, non-significant, Two-way repeated measure ANOVA;)”. For this reason, the analysis shown is the pooled measurement of the ipsi- and contralateral side.

*- In figure S2C, the authors show no correlation between the number of seizures and the number of targeted cells. How did the authors count this number of cells: is it per section or total number?* *It would be interesting to correlate the pS6 intensity of the targeted cells with the number of seizures, as in Nguyen et al., 2019 study.*

The quantification of the targeted cells per mouse is briefly described in the legend of **Figure S2C**. For each mouse we selected three non-consecutive (250-300 um apart from each other) targeted sections corresponding to the somatosensory region. After scanning through 12-15 sections per mouse we selected the section with the highest targeting and two additional sections, the first non-consecutive section more frontal and more caudal from this. From each of those sections we counted the total number of tdTomato+ cells using Fiji; those three values per mouse were then averaged to obtain an average number of targeted cells per mouse, represented on the y axis of **Figure S2C**. We now added a brief description of the method used to select the sections to count the targeted cells, to the methods section of the manuscript.

Our observations that there is no correlation between the number of targeted cells and seizure frequency is consistent with the Nguyen *et al*., 2019 study (**Figure S2C**). However, as mentioned by the reviewer, in Nguyen *et al*., 2019 a positive correlation between increasing levels of p-S6 intensity and seizures frequency, was shown. This could potentially indicate that increases in mTOR activity lead to a higher number of seizures. They are able to quantify this, since they titrate the amount of Rheb plasmid to obtain three separate groups with different levels of mTOR activity and an increasing number of seizures per day going from low levels to high levels of Rheb. In our case, we overexpress a mutated form of RHEB that cannot be inhibited by TSC (see **Figure 1** of the manuscript), therefore even transfecting low amounts of this mutant will already result in ceiling level of mTOR activity, as RHEB is the rate-limiting step, hence corresponding to the “high RHEB” group in the Nguyen study. Interestingly, within the “high RHEB” group of the Nguyen study, the variability in number of seizures per day is very high, and there the correlation between the pS6 levels and number of seizures appears to be no longer present (**Figure 4C** of [7]), as the mice with higher pS6 intensity show fewer seizures per day. Additionally, we see in our mouse model, that upon seizures the level of pS6 of the non-targeted neurons also goes up, which will make the relative increase in pS6 levels of the targeted cells lower. This can be explained with the finding that seizures in general induce rapid expression of RHEB transcripts and therefore further increase in mTOR pathway activation ([8]). Based on these results, we do not think that in our mouse model, the intensity of the pS6 levels is a reliable measure to correlate to the number of seizures.

*- In the LSL-Rheb and floxed-Rheb experiments (Figure 4), I suggest to separate the 2 experiments (or rearrange the Figure). Moreover, the authors mention a delayed onset of seizures between their groups and the group with a developmental deletion of Rheb, without showing the data: a survival graph such as in Figure S2A would be appreciated.*

We thank the reviewer for the suggestion. We do think that showing the two conditions close to each other in the same figure better fits the message, we rearranged the figure in a hopefully clearer way for the reader. Additionally, we added the survival graph for the LSL-Rheb group in new **Figure S3**.

*- In Figure 5C, the authors show improper cortical connectivity. As the targeted cells in ipsilateral region are misplaced: is there a correlation between the misplacement of the targeted cells (from IZ to layer 2-3) and their contralateral connexions (from deeper to upper layers)?*

We agree with the reviewer that this would be a very interesting element to assess. Unfortunately, in our experimental setup, we are not able to follow the axon of each individual cell to see where it projects. Hence, we do not have the precise answer to this question. However, in **Figure 5C** we show that most of the axons from the targeted cells terminate in deeper layers (5 and 6) and that the variability of these projections between sections as well as between mice is low, suggesting that even upon experimental variability in, for example, the amount of targeting or degree of migration deficit, the improper axonal connectivity is consistently present. Furthermore, we show with the optogenetic experiment that independently of where the projections go (L2/3 or L5), the synaptic contacts are functionally stronger.

- *A quantification of the bottons in the contralateral connexions may help assess whether synaptic vesicles have a higher density in Rheb-mutated cells, and potentially explain an increase of neurotransmitter release (Figure S3).*

We thank the reviewer for this suggestion. We have now performed Electron Microscopy on the brains of the RHEBp.P37L and the empty vector control targeted mice, for quantification of the axonal boutons in the contralateral projections. Confirming our confocal images, we find that the boutons on the contralateral side, on average are much larger in the RHEBp.P37L condition. Additionally, we find that the boutons in the RHEBp.P37L condition contain more mitochondria compared to the empty vector condition. Since the relative density of the vesicles in the boutons in the empty control versus the RHEBp.P37L condition appears to be similar, the mere fact that the boutons are larger can already explain an increase of neurotransmitter upon stimulation, as suggested by the reviewer. We have now added this data to **Figure S4** of the manuscript.

- *in the optogenetic experiments, it is not clear how the ChR2 were stimulated: in the ipsi-or the contra- lateral part? Was the ChR2 expression similar between control and Rheb-mutant? As the Rheb-mutant present potentially increased axonal length and number, it may be useful to normalize the amplitude recorded to this ChR2 expression.*

We used wide field optogenetic stimulation to directly stimulate the axons on the contralateral side. To quantify the ChR2 expression level, we have measured the mean fluorescent intensity of the ChR2-Venus signal on the ipsilateral side of these patched slices. We find that although there is a trend, the level of ChR2 is not significantly higher in the RHEBp.P37L condition, compared to the empty vector control (empty vector control, mean ± SEM = 1 ± 0.21; RHEBp.P37L, mean ± SEM = 1.24 ± 0.05; t(5) = 1.28; p = 0.26, unpaired t test). These results indicate that the ChR2 expression cannot fully explain the increased amplitude recorded upon ChR2 stimulation. We are unsure about normalizing the amplitude against the ChR2 expression because it is challenging to quantify the relationship between axonal photo current and transmitter release, although it is most likely non-linear. Therefore, we feel reporting normalized the amplitude does not bring further clarity to the results.

*- In the experiment of inducible Tetanus toxin, it would be clearer to separate both experiments: tamoxifen at P14 and tamoxifen after seizures (Figure 7). Moreover, it is not clear when the tamoxifen post-seizure stopped (Figure 7C): was it only 2 days of injections? In Figure 7E, it would be better to put an indicative age of the animal in day instead of the day after onset.*

We thank the reviewer for the suggestion, we now separated the experiments in **Figure 7** in three panels. All mice received 4 injections of tamoxifen, for both experiments presented in **Figure 7B** and **7C**. We rearranged the figure panel related to the tamoxifen post-seizures experiment (**Figure 7C**) to clarify this and also included a timeline on top of the graph similar to 7B. Additionally, we have included an indicative age of the animals in weeks, instead of days after onset, as suggested by the reviewer.

- *in the last experiment, the authors investigate the electrophysiological properties of targeted, non-targeted ipsi- and non-targeted contra-lateral neurons. However, they only patched neurons which migrated well to L2/3 (based on the fact that control patched neurons are in L2/3), but they also showed that the misplaced neurons (not migrating properly) kept their identity of L2/3 neurons (expressing Cux1). Therefore, it would have been interesting to assess electrophysiological properties of misplaced Rheb neurons to investigate their differences with the ones that migrated well. This should be discussed.*

We agree with the reviewer that it is interesting to look at the electrophysiological properties of targeted cells in the nodule. We now patched also those cells and, found that besides from a larger membrane capacitance (Cm), likely due to a bigger soma size, they behave electrophysiologically the same as the targeted cells in L2/3. These data are now presented in full in new **Figure S6** and discussed in the results section of the manuscript.

- *In this same experiment, they show that capacitance is increased in targeted Rheb cells compared to control empty vector. But this parameter seems to be similar (not significantly different) between targeted and non-targeted ipsilateral cells. Could the authors discuss this aspect? Could it be a non-cell autonomous effect? Indeed, looking at pS6 levels in Figure 1G and S1C, several non-targeted cells seem to have an increased pS6 levels, and a larger soma size.*

There is a trend for non-targeted cells ipsilaterally to show an increase in Cm. However, the average value is not significantly increased compared to both non-targeted cells or contralateral cells and does not differ from targeted cells. This makes the trend difficult to interpret in its biological relevance. Additionally, as the reviewer noticed, it does appear that some non-targeted cells in RHEB mice show a high baseline level of pS6 intensity that could go along with the trend towards the slight increase in the soma size. One explanation could indeed be a non-cell autonomous effect caused by the surrounding targeted cells or it might be a secondary effect caused by the presence of seizures. RHEB was first identified as a novel member of the Ras family of small GTP-binding proteins whose expression is rapidly induced upon seizures in the rat brain ([8]). Therefore, it is reasonable to observe a generalized (not confined to the targeted cells only) increase of the mTOR pathway upon epilepsy also in our mouse model. We now discussed more in details this aspect in the discussion.

- *in the discussion, the authors mention previous work from the Bordey's lab suggesting that SScx might be an epileptogenic area. It should be stated that other IUE models of mTOR pathway genes do have seizures from the SScx* *(Baek et al. 2015, D'Gama et al 2017; Lim et al., 2015; Hu et al., 2018, Ribierre et al., 2018, …).*

We thank the reviewer for pointing out this aspect. The work of Bordey is actually suggesting the contrary, emphasizing that the SScx might not be an epileptogenic area while the cingulate cortex is. In their experiments, targeting the SScx with the RHEBp.S16H construct does not cause development of spontaneous seizures whereas upon targeting the cingulate area, spontaneous seizures do occur ([9]). However, our study, as well as the other studies pointed out by Reviewer 2 where the SScx is targeted with other components of the mTOR pathway, prove the contrary. We included the correct citations in the discussion where this specific aspect is discussed.

- *Finally, it could be interesting to discuss how their result can be translated to human patients, according to the work of the lab of C. Cepeda.*

We included now in the discussion a more prominent section discussing the implication of our findings in relation to human findings, including results obtained from the C. Cepeda lab.

***Reviewer #3****: In a recent study (**Reijnders et al., 2017), the authors showed that over-expression of RhebP37L mutation leads to defects in neuronal migrations and seizures. In this study, Onori and colleagues further characterize the effect of this mutation in cortical malformations, seizures and axonal connectivity. The authors first show that the RhebP37L mutant is resistant to inhibition by the TSC complex and leads to increased mTORC1 activity, enlarged dysplastic neurons, cortical malformation and seizures. Using a clever approach that combines pharmacology and genetics, the authors that cortical malformation is neither necessary nor sufficient to induce epilepsy. Interestingly, the authors found that the RhebP37L mutation caused enhanced axonal connectivity and increased hyperexcitability. Moreover, blocking vesicle release was enough to prevent seizures in this model.

Overall, I find this to be a well-conducted and well-executed study that will be interested to many investigators in the field. The paper contains a lot of data and the authors should be lauded for their effort. In addition, the study provides strong evidence that cortical malformation and seizures can be dissociated and a potential mechanism (increased axonal connectivity) underlying the pathology associated with mTORopathies.*

We thank Prof. Costa-Mattioli for his kind words and we do hope that our study will contribute to a better understanding of cortical malformations and mTOR-related epilepsy.

 *I have a few questions/suggestions:*

*1. Fig. 1 and throughout the paper. The comparison should be performed between mutant RHEB (RhebP37L) and WT RHEB, instead of vector alone.*

We thank reviewer for this sharp observation. We have previously indeed performed such a comparison between mutant RHEB (RhebP37L) and WT RHEB ([10]). The main goal of that study was to evaluate the pathogenicity of two RHEB gene variants, including the RHEBp.P37L mutant, where we concluded that the variants were acting as dominant activating mutations causing an increase in the mTOR pathway activity. We showed that mice expressing one of the two variants (through *in utero* electroporation) had severe cortical migration defects associated with seizure development. In comparison, overexpression of the RHEB WT caused a milder migration delay, *without* the pronounced formation of a heterotopic nodule and only 20% of these targeted mice had seizures ([10]).

Building upon these findings, the main goal of our current study is to further understand *how* mTOR-dependent cortical malformations cause the development of epilepsy. Since 100% of the mice transfected with RHEBp.P37L mutant develop heterotopic nodules as well as seizures, we chose to compare that with the normal condition, in which none of the mice had migration delay and seizure. We feel this strategy is better controlled and therefore more suitable for our research questions.

Nevertheless, to confirm our previous findings, we have repeated the experiments as published in ([10]). Confirming previous results, overexpression of RHEB WT does cause a migration deficit, but much milder compared to the P37L mutant, with a more variable degree of migration across the cortex (as can be seen in *Rebuttal Figure 4* below, where bin 1 represents the outer layer of the cortex, and bin 10 the deepest layer of the cortex). Additionally, overexpression of RHEB WT, despite some neurons staying behind, does not result in the formation of a well-defined heterotopic nodule. Furthermore, none of the RHEB WT targeted mice showed any seizures (recorded until 2 months of age).

We now clarified in the manuscript our primary goal of this study to avoid any confusion on the use of the overexpressed RHEB WT as a control.

**Rebuttal Figure 4.** *Left*: representative picture of the targeted somatosensory cortex of a mouse targeted with the RHEB WT construct; dotted lines indicate the border of the intermediate zone (IZ, bottom) and delineate L2/3. ­­­Scale bars: 50 μm *Right*: distribution of tdTomato+ cells in the targeted somatosensory cortex of empty vector mice (N=5, 6 weeks old), RHEBp.P37L mice (N=5, 6 weeks old) and RHEB WT mice (N=6, 6 weeks old) calculated as % of targeted cells across the cortical layers, with bin1 indicating the upper layer 1 and bin 10 the lower layer 6. Data are presented as mean ± SEM.

*2. Fig. 2. Do the animals exhibit behavior seizures? Is survival affected in mice expressing RhebP37L in the brain?*

The RHEBp.P37L mice do show behavioral seizures that are recognizable by the loss of upright posture followed by a tonic-clonic phase with convulsions and twitching behavior. In our previous work ([10]), we showed a video where one of these epileptic episodes can be observed (Supplementary Movie 1 in the Electronic supplementary material at https://www.nature.com/articles/s41467-017-00933-6#Sec26).

We closely observed and performed EEG measurements on these mice up to 3 months of age and we never observed lethality within this timespan, however we cannot know for sure that the total lifespan of the RHEBp.P37L mice is not affected. It is possible that at a later stage survival might be reduced compared to wild type mice.

*3. Does the RhebP37L mutant alter mTORC2? If this is the case, does rapamycin treatment prevent the potential increase in mTORC2 activity?*

We assessed the effect of RHEBp.P37L overexpression in HEK cells on mTORC2 activation by looking at the phosphorylation levels of Akt on both Ser473 and Thr308, but did not detect a significant increase in mTORC2 activation when overexpressing the P37L mutant compared to the WT (see *Rebuttal Figure 5*).

**Rebuttal Figure 5:** *Left*: representative Western blot of lysates from HEK cells transfected with empty vector (EV) control, RHEB WT or RHEBp.P37L plasmid, in starvation or growth culturing conditions. *Right*: quantification of the western blot, showing the level of pAKT-S473 over total AKT for the different conditions.

*4. Fig. 4. The changes in migration depicted in this figure are very minor and statistics are required to support the author's argument.*

In **Figure 4** we are showing two complementary genetic experiments to support the hypothesis that the presence of the migration defect is not necessary nor sufficient to cause seizures. In one case we placed a Lox-STOP-Lox Cassette in front of RHEBp.P37L, to induce the expression only postnatally, and show that this condition does not cause any migration defect (all the cells are located in L2/3 and there is no heterotopic nodule since the mTOR pathway is not hyperactive during brain development). In the other case we placed LoxP sites around the RHEBp.P37L, for us to stop the expression postnatally, and as expected, this behaves exactly in the same way as the normal RHEBp.P37L construct, causing the formation of a heterotopic nodule. The pictures shown depict these two opposite conditions (absence *vs* presence of migration deficit) to illustrate the effect of these conditions. If with the minor difference in migration the reviewer is referring to the pictures *pre* and *post* tamoxifen, we are not claiming that tamoxifen is interfering with the cell migration. Tamoxifen injections are administered at a time point where the migration already occurred and the only thing happening upon tamoxifen administration is the activation (LSL condition) or reduction (Floxed condition) of mTOR hyperactivation. We now rearranged **Figure 4** to clarify the message and avoid any confusion on this aspect.

*5. Fig. 5. Again, WT mTOR should be used as control. Does expression of RhebP37L mutant leads to increased axon number (more than one axon) in vivo?*

As mentioned before in our reply to the first suggestion/question, we are evaluating how our mouse model (with the presence of a heterotopic nodule and seizures) differs from a normal WT mouse. The most important reason for this, especially for this anatomical aspect, is because we want to establish where in a control mouse the callosal projection neurons project with our experimental conditions and how our mouse model, with a clear heterotopic nodule as well as seizures, differs from that.

It has been shown before in literature that the mTOR pathway and RHEB play an important role in axonal outgrowth both *in vitro* and *in vivo*, with functional effects on neuronal network formation ([11–13]) and we expected our mutant to do the same. Since to be able to establish if the axons form more branches *in vivo* is technically challenging, we decided to assess this aspect *in vitro*. We believe that the results we obtained *in vitro* might be true also *in vivo*. An indication that points towards this assumption is the fact that we consistently observe axon terminals not only at the border of S1/S2 as would normally occur, but also in S1 and in the pyriform cortex (as shown in **Figure 5B**). Therefore, while we cannot establish at the single cell level if the mutant leads to more than one axon *in vivo*, we can reasonably say that, overall, expression of the P37L leads to longer axons and likely more branch terminals.

REBUTTAL REFERENCES

1.	Borrell V, Yoshimura Y, Callaway EM. Targeted gene delivery to telencephalic inhibitory neurons by directional in utero electroporation. J Neurosci Methods. 2005;143: 151–158. doi:10.1016/j.jneumeth.2004.09.027

2.	Nieto M, Monuki ES, Tang H, Imitola J, Haubst N, Khoury SJ, et al. Expression of Cux-1 and Cux-2 in the subventricular zone and upper layers II-IV of the cerebral cortex. J Comp Neurol. 2004;479: 168–180. doi:10.1002/cne.20322

3.	Cubelos B, Sebastián-Serrano A, Kim S, Redondo JM, Walsh C, Nieto M. Cux-1 and Cux-2 control the development of Reelin expressing cortical interneurons. Dev Neurobiol. 2008;68: 917–925. doi:10.1002/dneu.20626

4.	Huang Y, Song N-N, Lan W, Hu L, Su C-J, Ding Y-Q, et al. Expression of Transcription Factor Satb2 in Adult Mouse Brain. Anat Rec. 2013;296: 452–461. doi:10.1002/ar.22656

5.	Lodato S, Rouaux C, Quast KB, Jantrachotechatchawan C, Studer M, Hensch TK, et al. Excitatory Projection Neuron Subtypes Control the Distribution of Local Inhibitory Interneurons in the Cerebral Cortex. Neuron. 2011;69: 763–779. doi:10.1016/j.neuron.2011.01.015

6.	Rudy B, Fishell G, Lee SH, Hjerling-Leffler J. Three groups of interneurons account for nearly 100% of neocortical GABAergic neurons. Dev Neurobiol. 2011;71: 45–61. doi:10.1002/dneu.20853

7.	Nguyen LH, Mahadeo T, Bordey A. mTOR Hyperactivity Levels Influence the Severity of Epilepsy and Associated Neuropathology in an Experimental Model of Tuberous Sclerosis Complex and Focal Cortical Dysplasia. J Neurosci. 2019;39: 2762–2773. doi:10.1523/jneurosci.2260-18.2019

8.	Yamagata K, Sanders LK, Kaufmann WE, Yee W, Barnes CA, Nathans D, et al. Rheb, a Growth Factor- and Synaptic Activity-Regulated Gene, Encodes a Novel Ras-Related Protein. J Biol Chem. 1994;269: 16333–16339.

9.	Hsieh LS, Wen JH, Claycomb K, Huang Y, Harrsch FA, Naegele JR, et al. Convulsive seizures from experimental focal cortical dysplasia occur independently of cell misplacement. Nat Commun. 2016;7: 11753. doi:10.1038/ncomms11753

10.	Reijnders MRFRF, Kousi M, Van Woerden GMM, Klein M, Bralten J, Mancini GMSMS, et al. Variation in a range of mTOR-related genes associates with intracranial volume and intellectual disability. Nat Commun. 2017;8: 1052. doi:10.1038/s41467-017-00933-6

11.	Choi Y-J, Di Nardo A, Kramvis I, Meikle L, Kwiatkowski DJ, Sahin M, et al. Tuberous sclerosis complex proteins control axon formation. Genes Dev. 2008;22: 2485–95. doi:10.1101/gad.1685008

12.	Gong X, Zhang L, Huang T, Lin T V., Miyares L, Wen J, et al. Activating the translational repressor 4E-BP or reducing S6K-GSK3β activity prevents accelerated axon growth induced by hyperactive mTOR *in vivo*. Hum Mol Genet. 2015;24: 5746–5758. doi:10.1093/hmg/ddv295

13.	Nie D, Di Nardo A, Han JM, Baharanyi H, Kramvis I, Huynh T, et al. Tsc2-Rheb signaling regulates EphA-mediated axon guidance. Nat Neurosci. 2010;13: 163–72. doi:10.1038/nn.2477
