# Supplementary material for: RHEB/mTOR hyperactivity causes cortical malformations and epileptic seizures through increased axonal connectivity
Source: PLoS Biol. 2021 May 26;19(5):e3001279. doi: 10.1371/journal.pbio.3001279 (PMC8186814; doi:10.1371/journal.pbio.3001279)
Supplement: Attachment — Submitted filename: Response to the Reviewers.docx [file pbio.3001279.s022.docx]

**Response to the Reviewers:**

*Reviewer #1: The revised manuscript is well corrected and reconstructed. The responses are acceptable.*

We thank the reviewer for his positive feedback and we’re happy to hear that the responses were acceptable.

*Reviewer #2 (Stéphanie Baulac): The authors have very well addressed all questions raised by reviewer 2 and 3, and provided a large amount of new experiments.*

*I would like to congratulate the authors for this remarkable study, bringing important new insights to mTOR-related epileptogenesis.*

We are happy to hear that the reviewer is content with the new data provided and that we have addressed the questions very well. We thank Prof. Baulac for her positive feedback and her kind words on our work.

**Response to the editor:**

*1) ETHICS REQUEST: In the methods section of your manuscript, please include the full name of the IACUC/ethics committee that reviewed and approved the animal care and use protocol/permit/project license. Please also include an approval number (if different from the CCD number that you already provided).*

We have now added the following sentence:

“All animal experiments were conducted in accordance with the European Commission Council Directive 2010/63/EU (CCD project license AVD1010020172684), and all described experiments and protocols were subjected to ethical review (and approved) by an independent review board (IRB) of Erasmus MC.”

*2) DATA REQUEST: Please provide, as a supplementary file, the data that was used to generate each graph in your manuscript. When generating this file, please make sure to include a legend describing its contents. We will also need you to add a reference to this file in each figure legend (including supplmental). For example, you might say "the data underlying this figure can be found in S1_data. **For more specific information regarding this request, please see the notes below my signature.*

We have now added all the raw data requested by the editor and added in the figure legends where the data can be found.

*3) DATA REQUEST: Please provide original, uncropped and minimally adjusted images supporting all blot and gel results reported in an article's figures or Supporting Information files. **For more specific information regarding this request and a link to our guidelines, please see the notes below my signature.*

We have now added an uncropped and minimally adjusted image of the blot shown in Figure 1 in the Supporting Information files.

*4) We think that the title of your manuscript could be edited slightly to improve clarity and to note the relevance of your work to epilepsy. If you agree, we might suggest changing the title to something like "RHEB/mTOR-hyperactivity causes cortical malformations and epileptic seizures through increased axonal connectivity".*

We agree with the suggestion to change the title and have adjusted it in the manuscript.

*5) As you address these items, please take this last chance to review your reference list to ensure that it is complete and correct. If you have cited papers that have been retracted, please include the rationale for doing so in the manuscript text, or remove these references and replace them with relevant current references. Any changes to the reference list should be mentioned in the cover letter that accompanies your revised manuscript.*

While going through the manuscript and the figures, we found some typos in the figures (Figure 1 and Figure 8), which we have now adjusted. No changes were made in the reference list.

*ETHICS STATEMENT:*

*-- Please include the full name of the IACUC/ethics committee that reviewed and approved the animal care and use protocol/permit/project license. Please also include an approval number (if different from the number you already supplied).*

“All animal experiments were conducted in accordance with the European Commission Council Directive 2010/63/EU (CCD project license AVD1010020172684), and all described experiments and protocols were subjected to ethical review (and approved) by an independent review board (IRB) of Erasmus MC.”

*1) ETHICS REQUEST: In the methods section of your manuscript, please include the full name of the IACUC/ethics committee that reviewed and approved the animal care and use protocol/permit/project license. Please also include an approval number (if different from the CCD number that you already provided).*

We have now added the following sentence:

“All animal experiments were conducted in accordance with the European Commission Council Directive 2010/63/EU (CCD project license AVD1010020172684), and all described experiments and protocols were subjected to ethical review (and approved) by an independent review board (IRB) of Erasmus MC.”

*2) DATA REQUEST: Please provide, as a supplementary file, the data that was used to generate each graph in your manuscript. When generating this file, please make sure to include a legend describing its contents. We will also need you to add a reference to this file in each figure legend (including supplmental). For example, you might say "the data underlying this figure can be found in S1_data. **For more specific information regarding this request, please see the notes below my signature.*

We have now added all the raw data requested by the editor and added in the figure legends where the data can be found.

*3) DATA REQUEST: Please provide original, uncropped and minimally adjusted images supporting all blot and gel results reported in an article's figures or Supporting Information files. **For more specific information regarding this request and a link to our guidelines, please see the notes below my signature.*

We have now added an uncropped and minimally adjusted image of the blot shown in Figure 1 in the Supporting Information files.

*4) We think that the title of your manuscript could be edited slightly to improve clarity and to note the relevance of your work to epilepsy. If you agree, we might suggest changing the title to something like "RHEB/mTOR-hyperactivity causes cortical malformations and epileptic seizures through increased axonal connectivity".*

We agree with the suggestion to change the title and have adjusted it in the manuscript.

*5) As you address these items, please take this last chance to review your reference list to ensure that it is complete and correct. If you have cited papers that have been retracted, please include the rationale for doing so in the manuscript text, or remove these references and replace them with relevant current references. Any changes to the reference list should be mentioned in the cover letter that accompanies your revised manuscript.*

While going through the manuscript and the figures, we found some typos in the figures (Figure 1 and Figure 8), which we have now adjusted. No changes were made in the reference list.

*ETHICS STATEMENT:*

*-- Please include the full name of the IACUC/ethics committee that reviewed and approved the animal care and use protocol/permit/project license. Please also include an approval number (if different from the number you already supplied).*

“All animal experiments were conducted in accordance with the European Commission Council Directive 2010/63/EU (CCD project license AVD1010020172684), and all described experiments and protocols were subjected to ethical review (and approved) by an independent review board (IRB) of Erasmus MC.”
